# Continuity and Coordination of Care During Hospital‐To‐Home Transitions: Healthcare Professionals' Perspectives

**DOI:** 10.1111/jocn.17758

**Published:** 2025-03-26

**Authors:** J. W. M. van Grootel, R. J. Collet, J. M. van Dongen, M. van der Leeden, E. Geleijn, R. Ostelo, M. van der Schaaf, M. E. Major, S. Wiertsema

**Affiliations:** ^1^ Department of Rehabilitation Medicine Amsterdam UMC Location University of Amsterdam Amsterdam the Netherlands; ^2^ Amsterdam Movement Sciences, Ageing and Vitality Amsterdam the Netherlands; ^3^ Department of Rehabilitation Medicine Amsterdam UMC Location Vrije Universiteit Amsterdam Amsterdam the Netherlands; ^4^ Amsterdam Movement Sciences, Musculoskeletal Health Amsterdam the Netherlands; ^5^ Faculty of Science, Department of Health Sciences Vrije University Amsterdam Amsterdam the Netherlands; ^6^ Department of Epidemiology and Data Science Amsterdam UMC Location Vrije Universiteit Amsterdam Amsterdam the Netherlands; ^7^ Faculty of Health, Center of Expertise Urban Vitality Amsterdam University of Applied Sciences Amsterdam the Netherlands; ^8^ Faculty of Health, Department of Physical Therapy Amsterdam University of Applied Sciences Amsterdam the Netherlands

**Keywords:** aging in place, allied healthcare, experiences, hospital, older people, primary care, transitional care

## Abstract

**Aim:**

To gain insight into healthcare professionals' perceptions and needs regarding hospital‐to‐home transitions.

**Design:**

Qualitative phenomenological study.

**Methods:**

Hospital and primary care professionals participated in focus groups and interviews. Participants were recruited from a Dutch University hospital and from our networks between May and September 2023. Data were analysed using thematic analysis.

**Results:**

We conducted seven focus groups and twelve interviews. Three themes emerged: “Collaboration and information exchange between professionals”, “Coordination and continuity of care”, and “Interaction between professionals, patients, and families”.

**Conclusion:**

This study suggests that professionals would benefit from clear guidelines and arrangements for communication with colleagues to support care coordination and continuity. Collaboration and information sharing are essential for providing integrated, patient‐centred care. Additionally, involving patients and families in decision‐making regarding hospital‐to‐home transitions, in a way that considers their needs, is important for effective care.

**Implications:**

This study highlights the importance of clear communication and collaboration between professionals to ensure continuity of care. It emphasises the need for integrated care, where patients and families are actively involved without being overwhelmed.

**Impact:**

Transitions from hospital‐to‐home are often hindered by fragmented, non‐individualised care. Improved collaboration, clear coordination, and patient‐family involvement can address this. This research can positively impact professionals across different settings, policymakers, and advocacy groups aiming to improve integrated patient‐centred care.

**Patient or Public Contribution:**

The interview guide was developed with professionals who reviewed it and provided feedback. Professionals provided us with their lived experiences by participating in interviews and focus groups.

**Reporting Method:**

This study adhered to the COREQ guidelines.

**Trial Registration:**

N.A

**Summary**

- What does this paper contribute to the wider global clinical community?
  ○ The findings provide actionable insights that can inform practice and policy to optimise care coordination and improve patient outcomes in hospital‐to‐home transitions.
  ○ Researchers and professionals can use this information to develop transitional care interventions for patients with complex care needs.

## 1 Introduction

The aging population and the rising number of patients suffering from more than one disease (i.e., patients that often require care from one or more allied health professionals after discharge) underscore the importance of optimizing current transitional care practices (Sezgin et al. 2020; WHO 2023). Patients with complex care needs often face fragmented care, marked by a lack of coordination among healthcare providers, especially during transitions from hospital to home. This can lead to poorer health outcomes, such as unplanned readmissions. Evidence supports that patients with multiple diseases have a higher risk of unplanned readmissions and hospitalizations (Aubert et al. 2019; Rodrigues et al. 2022). These unplanned readmissions contribute to increased healthcare costs. In the Netherlands, for example, 40.9% of all healthcare costs were attributed to unplanned readmissions (Ribbink et al. 2019). This underscores the importance of optimizing care coordination during hospital‐to‐home transitions.

So‐called *transitional care interventions* have been developed to ensure effective coordination and continuity of care when patients transition from hospital to home or other destinations. These interventions include—but are not limited to—a combination of triage, discharge planning, involving families, appointing a case manager, and follow‐up (Tyler et al. 2023). Most of these interventions are unidirectional (i.e., led by nurses or medical doctors who are usually not in contact with primary care). However, many patients require more comprehensive care from multiple (allied) healthcare providers after discharge. Moreover, previous research has shown that current transitional care practices often remain task‐oriented despite the imperative for a multidisciplinary and person‐centred approach to enhance patient recovery (Allen et al. 2017; Dyrstad et al. 2015). However, sustainably implementing these interventions is challenging due to many individual, organisational, and systemic barriers (Collet et al. 2025). Examples of such challenges include the coordination of multiple stakeholders across settings, limited hospital bed availability, and insufficient financial resources. Moreover, involving healthcare professionals in designing transitional care interventions may facilitate implementation by addressing context‐specific challenges, fostering ownership, and aligning interventions with clinical practices to meet the needs of both patients and professionals (Fakha et al. 2023).

## 2 The Study

The objective of this phenomenological study is to gain insight into hospital and primary care professionals' beliefs, thoughts, perceptions, and needs regarding care‐coordination during hospital‐to‐home transitions. By examining their lived experiences, professionals can provide a deeper understanding of care coordination and continuity. This information can serve as a starting point for (re‐) designing an effective transmural care pathway.

## 3 Method

### 3.1 Design

This study used a descriptive phenomenological approach, based on Husserl's philosophy, to explore healthcare professionals' experiences, perceptions, and needs regarding care coordination and continuity during hospital‐to‐home transitions (Cudjoe 2023). Phenomenology can provide a thorough understanding of participants' lived experiences, their meanings, and their implications (Bliss 2016). In this study, phenomenology was used to explore healthcare professionals' experiences with care coordination and continuity, offering nuanced insights into key factors influencing hospital‐to‐home transitions. Since our research aims to understand how professionals perceive and navigate transitional care, rather than develop a theoretical framework on its processes, we were of the opinion that phenomenology was a more suitable methodological approach compared with other approaches (e.g., grounded theory). The Consolidated Criteria for Reporting Qualitative Research checklist was used to report findings in this study (Tong et al. 2007).

### 3.2 Study Setting and Recruitment

Healthcare professionals from a Dutch University hospital and primary care settings were purposefully sampled to participate in separate focus groups and/or in‐depth semi‐structured interviews. Hospital professionals were recruited through research team members working at the participating wards via email. Primary care professionals were recruited via regional professional allied healthcare networks, social media, and snowball sampling (Leighton et al. 2021). Recruitment continued until thematic saturation was achieved, i.e., when no new themes could be generated from the data.

### 3.3 Inclusion Criteria

Healthcare professionals were eligible if they (1) worked as a nurse, transfer nurse, physical therapist, occupational therapist, dietician, speech‐ and language therapist, medical doctor, practice nurse, or general practitioner, (2) worked in primary care within the Amsterdam region, or (3) worked in the departments of oncologic surgery, internal medicine, trauma surgery, or the intensive care unit of the participating hospital. These hospital departments were chosen because they include a wide variety of patients with complex care needs, for whom a structured allied healthcare pathway is typically lacking in the Netherlands. Furthermore, we feel that by selecting these four departments, a representative sample of healthcare professionals involved in transitional care is provided. Allied healthcare professionals, including physical therapists and dietitians, as well as nurses and medical doctors, were selected because they are responsible for referral and hand‐over information in hospital‐to‐home transitions in the Netherlands. Supporting Information File S1 provides a detailed description of the Dutch healthcare system.

### 3.4 Data Collection

The primary data collection method was focus groups, although participants could opt for an interview if scheduling or organisational constraints prevented focus group participation. Data collection took place between May and September 2023. Focus groups were pilot tested once (data not included). Hospital professionals participated in face‐to‐face focus groups at their respective wards, while semi‐structured interviews were conducted online via Microsoft Teams. Focus groups and interviews with primary care professionals were also held online. The topic guide was co‐created by members of the research team (SW, JvD, RC, and JvG), who have backgrounds in Clinical Epidemiology (RC), Human Movement Sciences (JvD and SW), and Physiotherapy (RC, JvG, and SW). During the study, questions about professionals' needs regarding handovers were added to the topic guide after the first focus groups revealed their relevance. Each focus group consisted of three discussion rounds. The first round aimed to introduce the topic through a warming‐up with general statements participants were invited to react to. During the second round, information was given about the first draft version of the new transmural care pathway, and participants were asked to brainstorm ideas. In the third round, a comprehensive summary of the focus group was provided to highlight the essential elements identified by healthcare professionals. Participants were invited to discuss the summary. A similar topic guide was used for the individual interviews (Supporting Information Files S2–S4).

The focus groups were facilitated by a mediator (JvG), an observer (SW, MM, or JvD), and a moderator who took field notes (RC). Two researchers (JvG and RC) completed a qualitative research course, and all researchers had extensive experience with this type of research. The interviews were conducted by JvG, who was already familiar with some of the participants. In addition to qualitative data, demographic information was collected, including age, gender, profession, ward, hospital/primary care setting, and years of working experience.

### 3.5 Data Analysis

Thematic analysis was applied to identify key themes and provide a structured understanding of their perspectives. (Bliss 2016; Braun and Clarke 2012). All focus groups and interviews were audio‐recorded and transcribed verbatim. MAXQDA 2022 (VERBI Software, 2021) was used for data analysis. Data were analysed using the following three steps (Braun and Clarke 2012):

1. Familiarisation with the data: transcripts were thoroughly read and re‐read to gain a deep understanding of participants' answers. Two researchers (JvG and RC) highlighted meaningful sentences related to care coordination and continuity (i.e., the phenomenon).
2. Generation of initial codes: meaningful sentences or issues relevant to the phenomenon were assigned general codes (JvG, RC, SW, and JvD).
3. Searching for, defining, and naming themes: the list of derived codes from step 2 was reviewed and grouped into categories based on similarities and characteristics. These categories were then synthesised into overarching themes. If needed, a definition of the theme was added.

Two researchers, RC and JvG, independently analysed the transcripts of the first two focus groups. RC and JvG discussed their codes and themes together. A third researcher (SW) checked the categories and themes to ensure consistency. Finally, JvG analysed the remaining transcripts. To explore the diversity of healthcare professionals' perspectives on care coordination during hospital‐to‐home transitions, themes derived from the focus groups and interviews were discussed during five reflexivity meetings. In these meetings, the analysis findings were shared and discussed with the whole research team to reflect on researchers' perspectives, assumptions, and potential biases, thereby enhancing the transparency and trustworthiness of the data analysis process (Creswell et al. 2007). Demographic data was entered into SPSS version 21, where categorical data were analysed for frequencies, and continuous data were analysed using descriptive statistics.

### 3.6 Ethical Considerations

The AUMC medical ethics committee provided a waiver for this study (METC 2023.0119). All participants provided written informed consent.

### 3.7 Rigour and Reflexivity

To ensure transparency in our content analysis, we provide access to our data repository upon request. To ensure the trustworthiness of the qualitative findings, four criteria—credibility, dependability, confirmability, and transferability—were incorporated into the study design (Busetto et al. 2020). Credibility was enhanced through independent open coding by two clinical researchers (RC and JvG) and involving a third researcher (SW and/or JvD) in phases two and three of the analysis. Dependability was ensured via member checking, where all participants received their focus group or interview transcript and were invited to verify its accuracy (Busetto et al. 2020). Probing and prompting questions were used to clarify and deepen participants' answers to promote confirmability. Finally, transferability was supported by regularly discussing the data analysis outcomes with the research team to ensure consistency and broader applicability.

## 4 Findings

Seven focus groups were conducted, four with hospital professionals and three with primary care professionals. On average, six (range 3–9) professionals participated per focus group. Twelve additional interviews were conducted with hospital professionals (*n* = 8) and primary care professionals (*n* = 4). Participants' mean age was 40 (SD 12) years and they had 15 (SD 12) years of working experience in their current profession. Most participants were female (*n* = 40, 75%). Detailed demographic information is presented in Table 1. Two professionals provided feedback on the member check; this feedback did not influence the results.

**TABLE 1:** baseline characteristics of healthcare professionals.

Demographic information	Total	Focus groups	Interviews
Age, years (mean ± SD)	40 ± 12	40 ± 12	40 ± 8
Female, *n*	40	30	10
Setting, *n*			
Primary care	20	16	4
Hospital	33	25	8
Profession primary care, *n*			
Physical therapist	7	7	0
Occupational therapist	4	4	0
Dietician	3	3	0
Speech‐ and language therapist	2	2	0
General practitioner	2	0	2
Practice nurse	2	0	2
Profession hospital, *n*			
Physical therapist	8	8	0
Occupational therapist	4	4	0
Dietician	4	4	0
Speech‐ and language therapist	3	2	1
Nurse	6	6	0
Transfer nurse	2	1	1
Medical doctor	6	0	6
Working experience, years (mean ± SD)	15 ± 12	17 ± 12	9 ± 8

*Note:* Transfer nurse: nurse who provides information about options such as home care or district nursing, requesting aids or (temporary) placement in a (geriatric) rehabilitation center, practice nurse: a position in general practitioner care in the Netherlands; the support person for the (general practitioner's) practice, There are two specialisations: somatic and mental health care.

Abbreviations: *n*, number; SD, standard deviation.

All focus groups lasted 60 min and were multidisciplinary, with at least four different disciplines represented in six of the seven focus groups. The median duration of the individual interviews was 23.59 (IQR 5.01) min. We identified one overarching concept, ‘communication’, containing the three following themes: (1) *Collaboration and information exchange between healthcare professionals*, (2) *Coordination and continuity of care*, and (3) *Interaction between professionals, patients, and families*.

**FIGURE 1:**
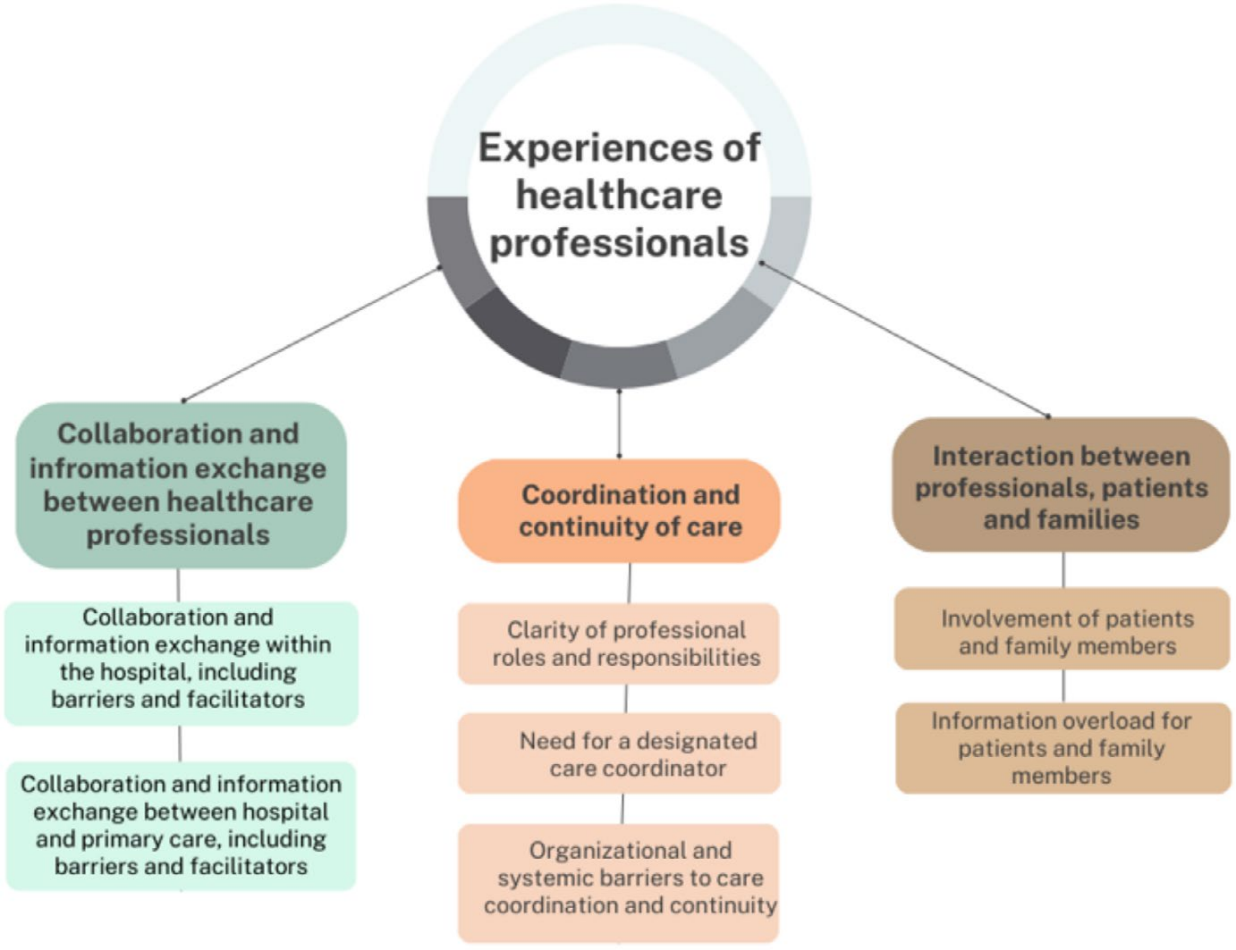
Overview of themes. [Colour figure can be viewed at wileyonlinelibrary.com]

The main text includes quotes illustrating these themes, with additional quotes provided in Supporting Information File S5. Figure 1 presents an overview of themes and subthemes. Across these themes, hospital and primary care professionals shared positive and negative experiences and perspectives on coordination and continuity of care during hospital‐to‐home transitions, as well as their views on areas for improvement.

### 4.1 Theme 1: Collaboration and Information Exchange Between Healthcare Professionals

This theme underscores the importance of information exchange during patient transitions from hospital to home. Two subthemes were identified: (1) *Collaboration and information exchange within the hospital, including barriers and facilitators, and* (2) *Collaboration and information exchange between hospital and primary care professionals*, *including barriers and facilitators*.

#### 4.1.1 Collaboration and Information Exchange Within the Hospital

Hospital professionals reported challenges in timely information exchange among ward colleagues regarding discharge planning. Discharges often feel sudden, leaving professionals and patients insufficiently prepared:

Sometimes I only hear about the discharge after the patient has already left the hospital, then I'm stuck with my back against the wall. If there are still things that need to be arranged for the patient, then that costs me a lot of indirect time (time for which the professional is not always paid). (Dietician, hospital)

The inefficient use of electronic patient records exacerbates these issues. Colleagues often fail to read notes, resulting in redundant questioning of patients about their medical history or home situation:

Sometimes the patients say “do you listen or read my (medical) records?” Because certain things are asked so often. (Nurse, hospital)

Furthermore, incomplete information about patients' home situations sometimes delays discharge planning. Hospital professionals indicated that information exchange was more effective with colleagues with whom they had direct relationships.

##### 4.1.1.1 Barriers and Facilitators Within the Hospital

Healthcare professionals generally experienced good communication and collaboration within hospital wards, facilitated by physical proximity:

Yes, and indeed, there is absolutely no threshold to approach someone. Yes, I actually think we work well together. (Physical therapist, hospital)

Trust in colleagues' expertise further supported collaboration. This is illustrated by the fact that they prepare themselves for patient visits using each other's notes in the electronic patient records. For example, physical therapists' assessments are pivotal in determining a patient's readiness for discharge:

If it is a challenge to guide the patient in the hospital‐to‐home transition, then we look at (the information from) the physical therapist. And even more so, we use the reports from the physical therapist to guide the patient (in the hospital‐to‐home transition). (Transfer nurse, hospital)

#### 4.1.2 Collaboration and Information Exchange Between Hospital and Primary Care Professionals

Information exchange between hospital and primary care professionals mainly occurs through referral letters, often unidirectional and delivered via patients. Less frequently, professionals call each other. Hospital professionals expressed a need for feedback on the quality and utility of these letters:

I'm also curious about what kind of information my primary care colleague wants (as far as information needed for an optimal hospital‐to‐home transition). (Occupational therapist, hospital)

In the referral letters, primary care professionals reported frequent incomplete or insufficient information about the patient's condition, course of recovery, and next steps to be taken after discharge. Additionally, they felt their responses to hospital colleagues were often disregarded.

I've had it fairly frequently that the discharge letter that I wrote was not even read. The patient sees that it (the discharge letter) just gets pushed aside and that conclusions are drawn without the information I provided. Then I think, yeah, why am I even doing this? (Occupational therapist, hospital)

Face‐to‐face interactions, such as multidisciplinary meetings, were viewed as ideal for exchanging information within professionals' own settings. For cross‐setting communication, phone calls or emails were preferred.

It is not commonplace yet, but multidisciplinary meetings should happen more often. It is nice to set up a treatment plan together. (Occupational therapist, primary care)

##### 4.1.2.1 Barriers and Facilitators Between Hospital and Primary Care

Belonging to a network of specialised professionals who regularly communicate with each other was identified as a facilitator for effective collaboration. Such networks facilitate communication between hospital and primary care professionals, patient referrals, and ultimately, the building up of strong interprofessional relationships:

The manner and ease of getting in touch with your colleague from another setting very much depends on the (professional) network within which the patients are referred. (Physical therapist primary care)

Conversely, professionals' availability and/or accessibility posed significant barriers to effective collaboration and information exchange. Misaligned work schedules and the lack of contact information often hindered timely communication:

Getting a hold of each other is also a challenge because everyone is also just working and treating patients. Sometimes colleagues complain about that when I don't answer. It's always a bit of a search for the best solution. (Occupational therapist, primary care)

### 4.2 Theme 2: Coordination and Continuity of Care

This theme highlights the need for effective care coordination and clear delineation of roles and responsibilities in hospital‐to‐home transitions. We identified three subthemes: (1) *Clarity of professional roles and responsibilities*, (2) *Need for a designated care coordinator*, and (3) *Organisational and systemic barriers to care coordination and continuity*.

#### 4.2.1 Clarity of Professional Roles and Responsibilities

Hospital and primary care professionals emphasised the lack of clarity regarding who is responsible for organising patients' discharge and follow‐up care. They stressed the importance of understanding which professionals participate in a patient's care:

It's common that you don't know (who is involved with the patient); you depend on what the patient tells you. And sometimes, I'm busy for over an hour looking through medical records for the names of involved professionals. It is helpful information to know (who is involved). (General practitioner, primary care)

While no formal agreement exists, many hospital professionals believed that nurses should take the lead in facilitating communication among ward professionals due to their close interaction with patients:

The nurses keep a very good record of their situations in the patients' files. They see the patient lying in front of them and have an impression of, “Oh, that patient can go home or that one can't”, and then they note it down. (Physical therapist, hospital)

Allied health professionals reported relying on nurses to compile and communicate to the rest of the team all information that is needed to prepare patients for discharge, such as treatment plans. Medical doctors retain responsibility for final discharge decisions, such as the moment and destination of discharge:

I feel that a portion of that responsibility (of the timing and planning of discharge) lies with the physician in charge; I believe they are the boss. (Physical therapist, hospital)

Hospital professionals also considered it their duty to assist patients with post‐discharge preparations, such as acquiring assistive devices:

If someone can't arrange something, then I do that with the computer at their bedside. If they can arrange it themselves, then I let them do it. That is a part of the treatment I give. (Physical therapist, hospital)

Finally, a lack of shared expectations among professionals was identified as a barrier to effective communication, with primary care professionals reporting that understanding hospital therapists' recovery expectations could guide when to contact them:

Expectations from hospital colleagues are necessary to get in contact (with them). If you know their expectations, for example, about the patient's status at discharge, you know when it doesn't go as it should. And then we know when to contact the hospital colleagues. (Physical therapist, primary care)

#### 4.2.2 Need for a Designated Care Coordinator

Professionals expressed a strong need for a coordinator to oversee hospital‐to‐home transitions. This coordinator should have a comprehensive understanding of patients' needs, facilitate communication between professionals, and organise follow‐up care. Hospital professionals suggested that the transfer nurse could fulfil this role, while a primary care physical therapist believed that this role would suit physical therapists well:

We have a role in screening how it is going (with the patient's recovery), so if it is not going well, for example, before the patient goes to a follow‐up appointment, then I sound the alarm. And (also) if I think other sorts of care are needed. (Physical therapist, primary care)

The designated coordinator would not need to provide direct medical care but should connect relevant professionals and streamline communication.

It is very important to have a regular contact person you can reach quickly and who makes communication (between organizations) easier; even if it is not the person you need to talk to, this contact should be able to forward you to the person you need to speak with. (Physical therapist, primary care)

Lastly, primary care professionals particularly valued the involvement of home nursing care services, as these providers regularly assess patients and refer them to appropriate allied health professionals.

#### 4.2.3 Organisational and Systemic Barriers to Care Coordination and Continuity

Hospital and primary care professionals identified several organisational and systemic barriers to effective care coordination and continuity. While recognising the importance of multidisciplinary collaboration within and between organisations, they acknowledged it was not always prioritised:

The follow‐up care clearly does not hold the same priority for everyone; engaging everyone in the department and maintaining a sense of proactiveness is a challenge. (Medical doctor, hospital)

Participants expressed frustration over the lack of standardised guidelines for communication and referral processes:

The discharge letter shouldn't be that way: I do it in my manner, and you do it in your manner. It would be great if that's possible (a uniform way of referring patients) because we are not there yet. (Medical doctor, hospital)

Primary care professionals also indicated they face challenges in maintaining continuity of care due to high turnover rates among general practitioners, who play a pivotal role in care coordination after discharge:

By the time I get my hands on one (a general practitioner), they're gone the following week. So that makes it difficult—an extra bump to have a good consultation. (Physical therapist, primary care)

Systemic barriers reported by professionals included the lack of financial support for interprofessional communication, which is not covered by health insurance. Professionals often perform these tasks outside regular hours, compromising their patient contact time:

We have to work together but it's not getting paid. (Physical therapist, primary care)

### 4.3 Theme 3: Interaction Between Professionals, Patients, and Families

This theme refers to the efficient involvement of patients and their family members as collaborators to optimise the hospital‐to‐home transition process. We identified two sub‐themes: (1) *Involvement of patients and family members* and (2) *Information overload for patients and family members*.

#### 4.3.1 Involvement of Patients and Family Members

Hospital professionals emphasised the importance of involving patients and their families from the start of the hospital‐to‐home transition process. Timely and clear communication about discharge planning enables patients and families to ask questions and prepare for post‐discharge care:

While you sit down (with the patient) and announce that you will talk about discharge, and you go through everything, it is much clearer. (Medical doctor, hospital)

However, hospital professionals mentioned that the extent of patient and family involvement should be balanced to avoid overburdening the healthcare team:

When it takes more time (involving patients and families in the hospital‐to‐home transition), yes, I think there will be resistance (from healthcare professionals on the ward), and more barriers will be raised. So, I think that's a good thing to keep in mind. (Medical doctor, hospital)

#### 4.3.2 Information Overload for Patients and Family Members

Both hospital and primary care professionals highlighted that the volume of information provided to patients at discharge often overwhelms them and does not seem to “stick” in their memory. This might result in delayed physical recovery, for instance, when patients forget that they were instructed to make a follow‐up appointment with a particular primary care professional or misplace the referral letter:

The communication (between hospital and primary care) goes via the patient, and the question is if that is actually good because the patient is not always adequate (to report medical information correctly to a healthcare professional). (Nurse, hospital)

Healthcare professionals further explained that patients are typically provided with large volumes of paperwork during their hospital stay, which can confuse them. Despite this, printed handover information remains essential to remind patients of essential steps for optimal recovery, such as scheduling follow‐up care. Moreover, professionals highlighted the issue of poorly timed discharge communication. Sudden discharges often leave patients and families feeling unprepared:

Sometimes you feel like there's a shortage of beds in the hospital and that you “oops” get a surprise patient dumped on your plate, and then I think, wow, they are not prepared! But other times everything is arranged, it varies a lot! (General practitioner, primary care)

## 5 Discussion

This study explored the beliefs, perceptions, and needs of healthcare professionals in hospital and primary care regarding coordination and continuity of care during hospital‐to‐home transitions. Three themes were identified: “Collaboration and information exchange between healthcare professionals”, “Coordination and continuity of care”, and “Interaction between professionals, patients, and families”. Considering these themes might be helpful when (re)designing a patient‐centered, multidisciplinary transitional care intervention aimed at facilitating coordination, collaboration, and adequate information exchange between professionals, patients, and their families. All the identified themes were ultimately connected to communication; therefore, in the subsequent discussion, we reflected on the topic of communication in each theme.

The first theme—*information exchange between healthcare professionals*—illustrates the challenges healthcare professionals face regarding information exchange. Within the hospital, discharge information is not always communicated to all professionals timely, sometimes resulting in delayed discharge. Between hospital and primary care, information exchange remains predominantly unidirectional. A recent systematic review that investigated handover challenges concerning safety and quality of health services corroborates our findings, as it identified a lack of communication as the main cause of reduced safety and quality of care (Raeisi et al. 2019). Additional challenges it identified included the absence of a discharge checklist, a lack of coordination, and poor task‐, space‐, and time management—several of which align with our findings (Raeisi et al. 2019). According to our results, primary care professionals often find the discharge letters unclear, while hospital professionals report hardly ever receiving questions about or feedback on their discharge letters. This indicates a misalignment among healthcare professionals across settings. Moreover, our finding that hospital professionals value knowing (how to find) their primary care counterparts responsible for follow‐up care aligns with findings from a previous study (Baxter et al. 2020). This study, exploring healthcare professionals' perceptions, highlighted that safe care transitions not only rely on knowing the patient well but also on strong interprofessional relationships across teams and settings (Baxter et al. 2020).

Theme 2—*Coordination and continuity of care*—illustrates the need for clear role definitions among healthcare professionals. Our study revealed a lack of standardised procedures for establishing contact with colleagues across different care settings, as well as limited time during work hours to plan (inter)organisational communication. These gaps often lead to insufficient communication about patients' rehabilitation plans, which might negatively impact hospital‐to‐home transitions. Healthcare professionals reported being unaware of each other's expectations regarding discharge letters, as well as who engages in the patient's care or who assumes responsibility during the hospital‐to‐home transition. Thus, lack of clarity hinders multi‐professional collaboration. To address these issues, our participants suggested appointing a designated case coordinator to oversee the hospital‐to‐home transition. Supporting this approach, a recent review identified essential strategies for effective case management, including creating personalised care plans and enhanced communication within multidisciplinary teams across diverse care settings (Braz et al. 2020). Further research should focus on multi‐professional communication to collaborate more effectively with each other and increase knowledge of each other's roles (i.e., how, what, and when to communicate).

Theme 3—*Interaction between professionals, patients, and families*—illustrates the importance of efficiently involving patients and families. Surprisingly, results revealed a discrepancy in the perspectives of hospital and primary care professionals: primary care professionals rarely mentioned patient and family involvement during the focus groups or interviews, whereas hospital professionals emphasised the importance of involving patients and family members. This difference can be explained by the fact that patients' home situations are vital information in making a treatment plan for hospital professionals, while primary care professionals focus more on improving mobility, which may necessitate less family involvement. Furthermore, a challenge we identified was the loss of essential information during patient discharge. This finding aligns with several prior studies reporting that the information provided can be overwhelming for patients and often poorly timed Agerholm et al. (2023); Allen et al. (2018); van Grootel et al. (2024). Engagement of patients and family members was found to be one of the essential components of effective hospital‐to‐home transitions in a previous study Naylor et al. (2017).

### 5.1 Strengths and Limitations

This study included a diverse sample of 53 healthcare professionals with varied expertise, including allied healthcare professionals, nurse practitioners, and medical doctors from both hospital and primary care settings. The additional interviews we performed after the focus groups provided more in‐depth information, and thematic saturation was reached. Consequently, our findings regarding allied health needs in the hospital‐to‐home transition may apply to a broad population. This study is conducted with methodological rigour, contributing to our results' transparency. However, there are also limitations to our study. First, all hospital healthcare professionals who participated in this study worked in an academic hospital; no conclusions can be drawn from general hospital professionals, affecting our results' generalisability. Second, the composition of our sample may influence the breadth of perspectives on hospital‐to‐home transitions, as dietitians have different levels of involvement in care transitions compared to, for example, nurses and medical doctors. This should be considered when interpreting the results in other healthcare contexts. Third, most information was collected from hospital‐ and primary care physical therapists. Less was collected from professionals from other allied health disciplines; this could be due to our recruitment strategy and may have resulted in unbalanced results. Physical therapists occupy a large share of the Dutch healthcare system. To provide perspective, in 2023, 36.166 physical therapists and 5.250 dieticians were registered in the Netherlands (CIBG 2023; PPN 2023).

### 5.2 Recommendations for Further Research

Future research should focus on translating and implementing our findings into the daily practices of hospital and primary care professionals. One area for investigation is the use of digital communication tools to enhance handovers between healthcare professionals. Research could also explore the effectiveness of uniform templates for handovers, as research suggests that they have the potential to improve consistency and clarity in communication (Bally et al. 2023; Fitzpatrick 2023; Rammant et al. 2021). Next to that, the integration of a case coordinator into daily practice could be explored as a possible means to avoid placing extra time demands on healthcare professionals or increasing costs for healthcare organisations (Thys et al. 2024).

### 5.3 Implications for Policy and Practice

The three themes are interconnected, with improvements in one area positively impacting the others. For instance, enhancing communication in information exchange (Theme 1) can lead to more effective coordination of care (Theme 2), because more accurate information allows professionals to align their actions. This, in turn, improves interactions between professionals, patients, and families (Theme 3). By addressing each of these themes, the overall quality of hospital‐to‐home transitions can be significantly improved.

Optimising collaboration between patients, families, and healthcare professionals can be achieved through strategies like regular multidisciplinary meetings, structured family involvement in care planning, and tailored communication tools (e.g., shared decision‐making aids, care diaries). Additionally, digital communication tools could further improve collaboration by enabling seamless information sharing across hospital and primary care settings (Chartrand et al. 2023; Fitzpatrick 2023). Moreover, our findings support the coordination of referrals within specialised care networks van Grootel et al. (2024). These strategies promote clear communication and ensure that all stakeholders are involved in the care plan.

## 6 Conclusion

This study aimed to gain insight into healthcare professionals' thoughts, beliefs, perceptions, and needs regarding hospital‐to‐home transitions. The findings suggest that future hospital‐to‐home transitions could be improved by optimising interprofessional communication (e.g., multidisciplinary, and interactive communication that comprehends more than a one‐way handover). Moreover, more clarity is needed for healthcare professionals regarding the question of ‘who does what?’ during hospital‐to‐home transitions and information should be presented in a way that is comprehensible for patients and families.

## Author Contributions

**J. W. M. van Grootel:** conceptualization, methodology, data collection, software, validation, formal analysis, writing original draft, visualisation, project administration, writing review and editing. **R. J. Collet:** conceptualization, methodology, software, formal analysis, validation, data collection, writing review and editing, visualisation. **J. M. van Dongen:** conceptualization, validation, data collection, writing review and editing, visualisation. **E. Geleijn, , M. E. Major, R. Ostelo and M. van der Schaaf:** conceptualization, writing review and editing. **M. van der Leeden:** conceptualization, validation, writing review and editing. **S. Wiertsema:** conceptualization, methodology, data collection, writing review and editing, investigation, project administration, supervision.

## Ethics Statement

The medical ethics committee of the Amsterdam University Medical Centers provided a waiver for this study (METC 2023.0119).

## Conflicts of Interest

The authors declare no conflicts of interest.

## Supporting information


**Data S1.** Supporting Information.

**Appendix 1.** The Dutch healthcare system.

**Appendix 2.** Focus group guide hospital professionals.
**Appendix 3**. Focus group guide primary care professionals.
**Appendix 4**. Topic guide interviews healthcare professionals.
**Appendix 5**. Additional quotes.

## Data Availability

The data that support the findings of this study are available on request via DataVerseNL: https://doi.org/10.34894/GBGDDL.
