# Supplementary material for: Continuity and Coordination of Care During Hospital‐To‐Home Transitions: Healthcare Professionals' Perspectives
Source: J Clin Nurs. 2025 Mar 26;35(5):2429–39. doi: 10.1111/jocn.17758 (PMC13068181; doi:10.1111/jocn.17758)
Supplement: Supplementary file 3 — Appendix 2. Focus group guide hospital professionals. Appendix 3. Focus group guide primary care professionals. Appendix 4. Topic guide interviews healthcare professionals. Appendix 5. Additional quotes. [file JOCN-35-2429-s001.docx]

**Appendix 2: focus group guide hospital professionals**

Introduction & informed consent

What is TULIP in brief

3 rounds of information exchange

Evaluation

-------------------------------------------

I work well with colleagues from other disciplines both within our department and within our hospital? Agree/Disagree and why?

The current transition from hospital to home is going well in our department. Agree/Disagree and why?

I feel it is my job to properly prepare patients for discharge. If not, who is?

What tasks do you think there are to do around discharge and who is responsible for this task? (1 task per post-it)

What do you think is already going well around dismissal (green post-it)? What do you think is going less well (red post-it)?

Some information about the research project

Our care is currently structured to allow for development and implementation of this new care, as described here. Agree/disagree and why?

What do you need to know about your patient before you can determine discharge destination?

Is there a need for a tool to screen patients for what they need in primary care? (Think outside your own discipline as well)

Where do you think the biggest challenge lies for first-line colleagues in patients with complex care needs?

-----------------------------------------

Evaluation

**Appendix 3: focus group guide primary care professionals**

Introduction & informed consent

What is TULIP in brief

3 rounds of information exchange

Evaluation

-------------------------------------------

I work well with colleagues in my discipline within my setting and outside my setting? Agree/Disagree and why?

The transfer of patients from hospital to primary care is going sufficiently within my discipline of care? De overdracht voor patiënten van het ziekenhuis naar huis/eerste lijn loopt goed binnen mijn discipline? Agree/Disagree and why?

What goes well around communication about this patient group? Consider communication with other disciplines or with the hospital (put in the chat with a +) What do you think is going less well (put in the chat with a -)

Information about the research project

What are reasons for contacting or not contacting a hospital colleague? What does it take to make contact? What would be a solution to improve this? In what kind of patients do you often contact? (Specific condition?)

What kind of information would you like to receive from your hospital colleague (put in the chat)

What do you think is still missing in terms of collaboration within paramedic care after complex hospitalization? And how could we improve this?

How do you think the patient is prepared by the hospital colleague for a transition to primary care?

Evaluation

**Appendix 4: Topic guide interviews healthcare professionals**

Introduction

Collaboration with other professionals

Communication with other professionals

Hospital colleagues

Primary care colleagues

Rehabilitation centers

Digital communication platform

C-boards

Triage/screening tool

Knowing about transition

Who is in charge

Who is involved

Areas for improvement

Examples

**Appendix 5: Additional quotes**

| **Subtheme** | **Quote** |
| --- | --- |
| Collaboration and information exchange within the hospital | “Well, everyone writes something in the records about the home situation because that is incredibly important for us as transfer nurses. But it is not uniform; I must carefully read through all the patient files (written by multiple professionals). It is really regrettable that we continually have to ask the same questions.” (Transfer nurse, hospital) |
|  | “Your direct coworkers whom you can walk right up to and quickly make contact and arrangements with. That is very important: immediate accessibility (with other healthcare professionals).” (Physical therapist, hospital) |
| Collaboration and information exchange between hospital and primary care professionals | “You never hear anything back from primary care colleagues. They don’t know what the patient has been through here, and that’s not to be expected (that primary care professionals know what the patient has been through in the hospital).” (Physical therapist, hospital) |
|  | “But you can hardly blame them (for not asking questions to hospital colleagues), but there are gaps to fill in: you rarely hear anything back.” (Physical therapist, hospital) |
|  | “I greatly desire information about what has happened and has been done with the patient and what he has endured. There seems to be a lot of resistance (from the hospital colleagues) to communicate all of that.” (Physical therapist, primary care) |
|  | “There is definitely profit to be made there if they (the patients) are doing just a little bit well, then suddenly things can go fast, and that's because of the bed pressure. Then there is very little time, and that could be better.” (Physical therapist, hospital) |
|  | “My experience is that the medical specialists are poorly approachable.” (Physical therapist, primary care) |
| Need for clear professional roles and responsibilities | “We sometimes don’t know who should be calling who and/or who is responsible for that part.” (Nurse, Hospital) |
|  | “And now everyone is working separately. And at a certain moment, it comes together.” (Occupational therapist, hospital) |
| Organizational and systemic barriers to care coordination and continuity | “Yeah, collaborating is, of course, incredibly important, I think. So, I am always open to that sort of initiative (to work together).” (Practice nurse, primary care) |
|  | “What I’ve noticed is that there is no agreement over how we exchange information and how we can reach each other.” (Physical therapist, primary care) |
| Need for a designated coordinator | “Yes, they (home-based nursing care staff) keep an eye on things. They indicate (what is needed in the patient’s recovery) and I think they make the transition easier.” (General practitioner, primary care) |
| Information overload for patients and family members | “I don’t know if they just didn’t listen well, but some people don’t even know they need to request physical therapy and only realize it after two weeks.” (Physical therapist, primary care) |
|  | “I think that patients prefer a physical letter. Then, reading goes faster, and they can give it to other healthcare professionals more easily.” (Dietician, hospital) |
